# Green Composites Based on *Hedysarum coronarium* with Outstanding FDM Printability and Mechanical Performance

**DOI:** 10.3390/polym14061198

**Published:** 2022-03-16

**Authors:** Roberto Scaffaro, Emmanuel Fortunato Gulino, Maria Clara Citarrella, Andrea Maio

**Affiliations:** Department of Engineering, University of Palermo, Viale delle Scienze, 90128 Palermo, Italy; emmanuelfortunato.gulino@unipa.it (E.F.G.); mariaclara.citarrella@unipa.it (M.C.C.)

**Keywords:** *Hedysarum coronarium*, sulla, polylactic acid, FDM, 3D printing, biocomposites, composites, mechanical properties, biopolymers, natural filler

## Abstract

The addition of natural scraps to biodegradable polymers has gained particular interest in recent years, allowing reducing environmental pollution related to traditional plastic. In this work, new composites were fabricated by adding 10% or 20% of *Hedysarum coronarium* (HC) flour to Poly (lactic acid) (PLA). The two formulations were first produced by twin screw extrusion and the obtained filaments were then employed for the fabrication of composites, either for compression molding (CM) or by fused deposition modeling (FDM), and characterized from a morphological and mechanical point of view. Through FDM it was possible to achieve dense structures with good wettability of the filler that, on the contrary, cannot be obtained by CM. The results indicate that the filler effectively acts as reinforcement, especially for FDM composites. The most remarkable enhancement was found in the flexural properties (+100% of modulus and ultimate strength), followed by tensile resistance and stiffness (+60%) and impact strength (+50%), whereas a moderate loss in tensile deformability was observed, especially at the highest loading. By adding HC to the polymeric matrix, it was possible to obtain a green, high-performance, and cost-effective composite, which could find applications for the fabrication of panels for furniture or the automotive industry.

## 1. Introduction

The addition of agricultural or marine waste to biodegradable polymers has gained particular interest, in recent years, for the fabrication of ‘green’ composite materials with high mechanical performance [1,2,3,4,5,6]. Green composites, in fact, may play a key role in the reduction of environmental impact related to plastic [7,8,9,10]. Moreover, the addition of waste biomasses to a polymeric matrix may reduce the costs of the final product.

Polylactic acid (PLA), polycaprolactone (PCL), polybutylene adipate terephthalate (PBAT), cellulose, and starch-based ones are some of the most commonly employed biopolymeric matrices for green composite production [1,7]. Several studies have shown the suitability of PLA to be used together with a natural filler to produce green composites [11]. By adding organic filler to PLA, in fact, it is possible to enhance its mechanical performance [1,8,12,13,14,15] and contextually accelerate its biodegradability [7,8]. A great variety of plant-based biomasses, typical of the Mediterranean area, may potentially help in achieving both goals, and allow obtaining biodegradable polymer-based composites that could find relevant applications in many fields. *Posidonia oceanica* leaves (POL), for example, were added to different biopolymeric matrices, in order to investigate their structure–properties relationships. The outcomes revealed that the addition of POL enhanced the mechanical properties of polymeric matrices [13,14,16] and accelerated their degradability [17,18]. Green composites prepared by adding two different amounts (10% and 20%) of *Opuntia Ficus Indica* flour to PLA showed an increase of elastic modulus on increasing the filler content [12]. Recently, 20 wt% of short banana fibers were added to PLA. These bio-composites displayed a significant improvement in mechanical properties if compared with the neat polymer [19].

*Hedysarum coronarium* (HC) is a fodder, watery herbaceous plant, rich in soluble sugars, and consisting in a very dense and structured root system and a branched, hollow, and fistulous stem [20,21]. HC, moreover, is considered a weed grass, due to its tendency to accumulate in countryside areas. Sometimes, this causes significant problems, since it needs to be disposed. In a previous study, innovative green composites were produced by compression molding and fused deposition modeling (FDM) by adding 5%, 10%, 15%, and 20% of HC flour to a starch-based biodegradable polymer. Mechanical tests showed that the filler effectively acted as reinforcement of the matrix [6].

To the best of our knowledge HC has never previously been used in combination with PLA, in order to prepare green composites.

Typically, thermoplastic-based composites containing natural filler are produced by injection molding, compression molding, or extrusion [22]. Recently, FDM has been investigated as an alternative method of green composite processing, due to the possibility of reducing production times and costs, while contextually creating extremely elaborate geometries [6,11,23,24,25,26,27]. These characteristics make FDM one of the most promising techniques for the production of green composites-based items [27].

**Figure 1:**
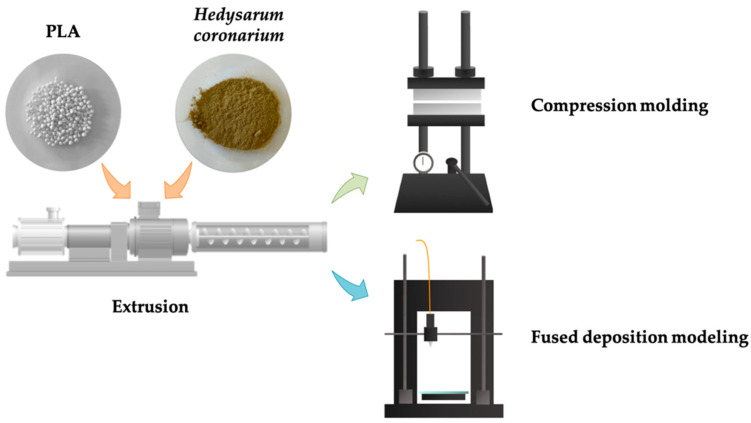
Schematic representation of the process.

In this study, new green composites were fabricated by adding 10% or 20% of HC flour to PLA. The two formulations were produced by extrusion, and the obtained filaments were then employed for the fabrication of composites by both CM and FDM, as depicted in Figure 1. Morphological and mechanical characterization was carried out on neat PLA and the obtained green composites. By adding HC to the polymeric matrix, it was possible to obtain a green and cost-effective composite that can be processed as a neat polymer, without performing any particular purification/chemical treatment, and employed for the fabrication of panels for furniture or automotive uses, in full compliance with zero-waste and circular economy guidelines.

## 2. Materials and Methods

### 2.1. Materials

PLA 2003D (density = 1.25 g/cm^3^; melt flow index = 6 g/10 min; 4.3% content of D-lactic acid monomer) was purchased from NatureWorks^®^ (Minnetonka, MN, USA). HC (*Hedysarum coronarium* L., syn. Sulla coronaria [L.] Medik. Species Sparacia) used in this study was collected from ‘Azienda Agricola Alberto Lo Dico’ Petralia Soprana (PA), Sicily and successively washed and dried in a vacuum oven (NSV9035, ISCO, Milan, Italy) at T = 40 °C for 3 days. From a visual inspection, it was possible to notice that the received biomass was made up entirely of *Hedysarum coronarium*. HC dried stems showed a Young’s modulus of 1545 MPa, a tensile strength of 23.3 MPa, and an elongation-at-break of 20.3%. HC flour, obtained by grinding the entire received biomass together (stems, leaves, and flowers), displayed an average density of 1.6 g/cm.

Preparation and characterizations of materials was performed at 20 °C and 50% relative humidity.

### 2.2. Preparation of Composites

In this study, the whole plant was ground as received, in order to optimize production time and costs.

Dried HC was ground for 3 min and then sieved. A sieving fraction under 150 μm was selected, in order to obtain particles of a size suitable for the 3D printer (Next Generation, Sharebot, Nibionno, Italy), which, therefore, do not cause obstruction to the nozzle. Prior to processing, in order to prevent polymer hydrolytic scission during processing, HC flours and PLA pellets were dried overnight in a vacuum oven (NSV9035, ISCO, Milan, Italy) at 40 °C and 90 °C, respectively. According to previous studies [6,23], PLA composites containing 10 wt% and 20 wt% of HC (label as PLA/HC10 and PLA/HC20) were prepared by extrusion in a Polylab single-screw extruder (Haake Technik GmbH, Vreden, Germany; L/D = 25; D = 19.05 mm), operating at 20 rpm screw speed and 160–170–180–190 °C temperature profile. The extrudates were drawn with the help of a conveyor belt system (take-up speed = 5.5 m/min), in order to obtain filaments with a diameter suitable for the printer (1.75 mm). Pure PLA was also extruded for comparison.

Compression-molded samples (CM) were obtained using a laboratory press (Carver, Wabash, IN, USA) at 190 °C and 180 bar for 3 min. The samples were finally cut into specimens of appropriate geometry for further characterizations: 60 mm × 10 mm × 2 mm for tensile tests, 40 mm × 10 mm × 2 mm for flexural tests, and 80 mm × 10 mm × 2 mm for impact tests.

As regards fused deposition modeling samples (FDM), they were first designed using a CAD Solid Edge 2019^®^ software (Plano, TX, USA), and the STL files produced were then elaborated on Simplify3D^®^ software (Cincinnati, OH, USA) to obtain the gcode files. Samples of each formulation were then 3D printed in an appropriate geometry (60 mm × 10 mm × 2 mm for tensile tests, 40 mm × 10 mm × 2 mm for flexural tests, and 80 mm × 10 mm × 2 mm for impact tests). FDM operating parameters are reported in Table 1. Nozzle temperature was chosen after some trials, aiming to avoid nozzle obstructions and to obtain good printability performance. Other parameters were chosen based on the scientific literature [6,23,28,29,30]. In particular, a 100% infill rate and a rectilinear infill pattern with ±45° raster angle were chosen, in order to optimize mechanical properties [26]; 30 mm/s printing speed was chosen as a compromise solution between better properties and higher production rate.

**Figure 2:**
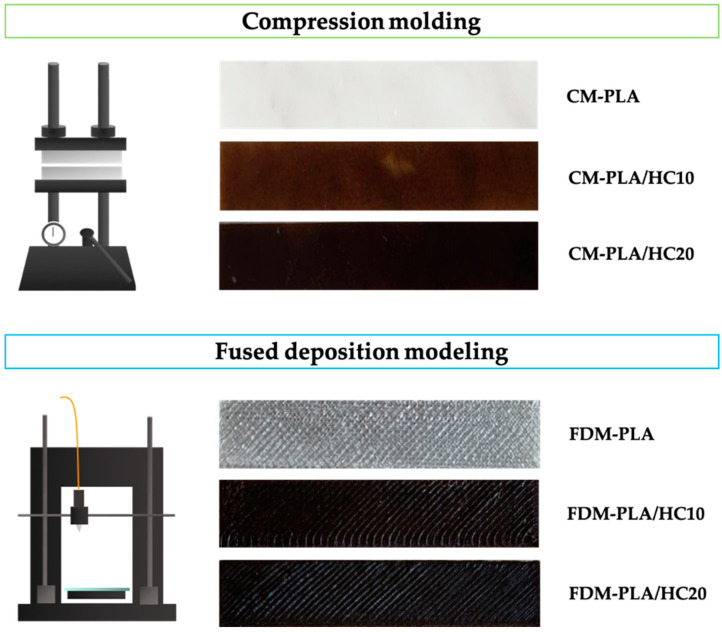
Digital photographs of CM and FDM obtained samples.

Sample formulations and their code name are reported in Table 2. Moreover, some representatives of obtained CM and FDM samples are shown in Figure 2.

### 2.3. Morphological Analysis

The morphology of CM and FDM samples was observed using a scanning electron microscope (Phenom ProX, Phenom-World, Eindhoven, The Netherlands), with optical magnification range of 20–135×, electron magnification range of 80–1.3×10^5^, maximal digital zoom of 12×, and acceleration voltages of 15 kV. The microscope was equipped with a temperature controlled (25 °C) sample holder. The samples were fixed on an aluminum stub (pin stub 25 mm, Phenom-World, Eindhoven, The Netherlands) using glued carbon tape.

### 2.4. Mechanical Characterization

Tensile properties of the samples were investigated using tensile tests, carried out using a laboratory dynamometer (mod.3365, Instron, Norwood, MA, USA) equipped with a 1 kN load cell. The tests were performed on rectangular shaped specimens, as described above. The measurements were performed by using a double crosshead speed: 1 mm min^−1^ for 2 min and 50 mm min^−1^, until fracture occurred. The grip distance was 30 mm, whereas the sample thickness was measured before each test. Eight specimens were tested for each sample.

Tensile modulus was calculated as the slope of the initial linear portion of the stress–strain curve, tensile strength (TS) and elongation at break (EB) were calculated as the maximum stress and the maximum strain before failure. Toughness (*k*) was calculated as the area under the stress–strain curve, Equation (1):

(1) $$k=\int_{0}^{\varepsilon_{b}}\sigma \left(\varepsilon \right)d\varepsilon$$

where ε is the strain, $\varepsilon_{b}$ is the strain upon failure, σ is the stress, and k represents the energy absorbed from each material (per unit of volume) without breaking.

Flexural tests in three-point bending mode were performed according to the ASTM D790 standard, by using the same apparatus under the same environmental conditions, aiming to measure the flexural modulus (FM) and flexural strength (FS). Flexural strength (FS) was calculated according to Equation (2):

(2) $$\mathrm{FS}=\frac{3F_{\max}L}{2bh^{2}}$$

where $F_{\max}$ is the maximum applied force expressed in *N*, *L* is the span between the supports in mm, whereas *b* and *h* are, respectively, the width and thickness of the specimen, expressed in mm. Flexural break (FB) is the maximum flexural strain, calculated according to Equation (3):

(3) $$\mathrm{FB}=\frac{6hd_{\max}}{L^{2}}$$

where $d_{\max}$ is the maximum deflection (mm). Flexural modulus was calculated as the slope of the linear portion of the load deflection curve. Flexural toughness was calculated in the same manner as indicated for tensile testing.

Impact tests in Charpy mode were performed using a pendulum model 9050 by CEAST (Italy), according to the ASTM D6110 standard, to evaluate impact strength (IS).

## 3. Results and Discussion

**Figure 3:**
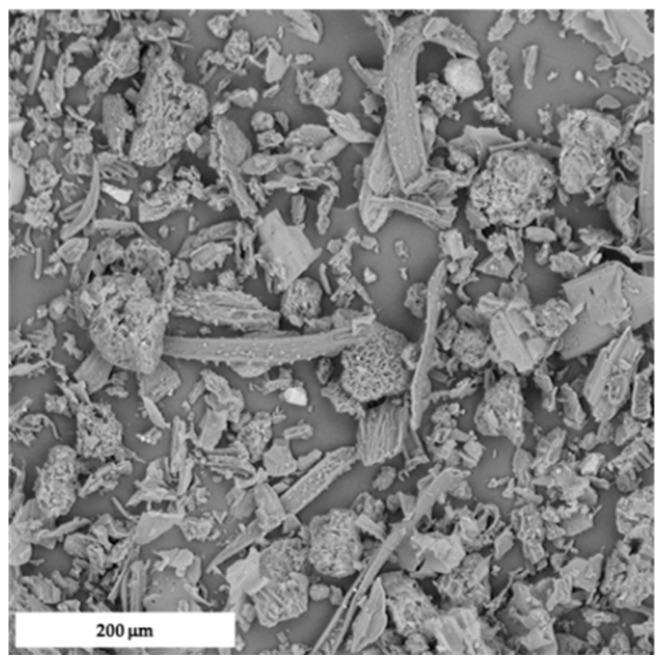
SEM micrograph of HC.

Morphology of HC particles and composites were analyzed by SEM, and a relevant micrograph of the natural organic filler is shown in Figure 3.

HC filler was characterized by the coexistence of fibers, platelets, and globular microparticles, possibly due to the presence of stems, leaves, and flowers. Indeed, this heterogeneity is supposed to impart benefits to the mechanical performance of the resulting composites [6], whereby the different types of element in HC are able to exert a synergistic effect, as already reported in several studies focused on hybrid composites containing either micro- or nano-sized fillers [31,32,33].

**Figure 4:**
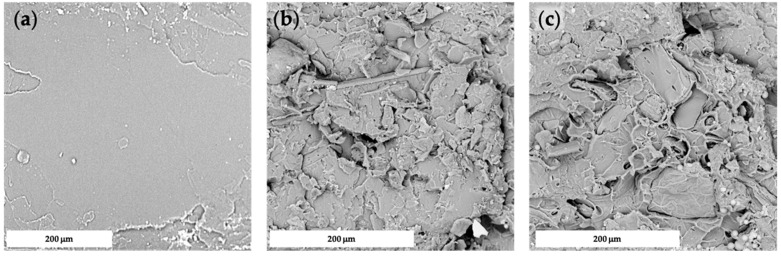
SEM micrographs of fractured cross-section of CM_PLA (**a**), CM_PLA/HC10 (**b**), and CM_PLA/HC20 (**c**).

**Figure 5:**
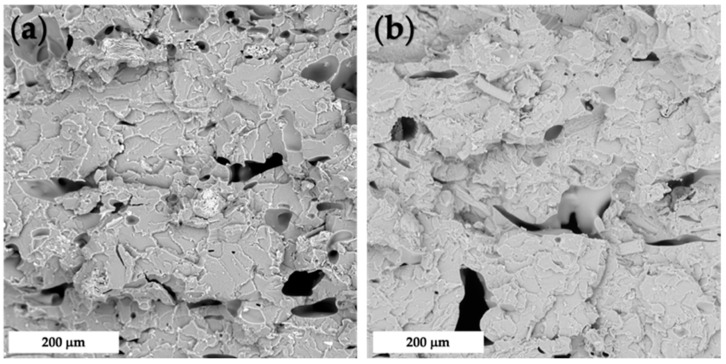
SEM micrograph of fractured cross-section of FDM_PLA/HC10 (**a**) and FDM_PLA/HC20 (**b**).

**Figure 6:**
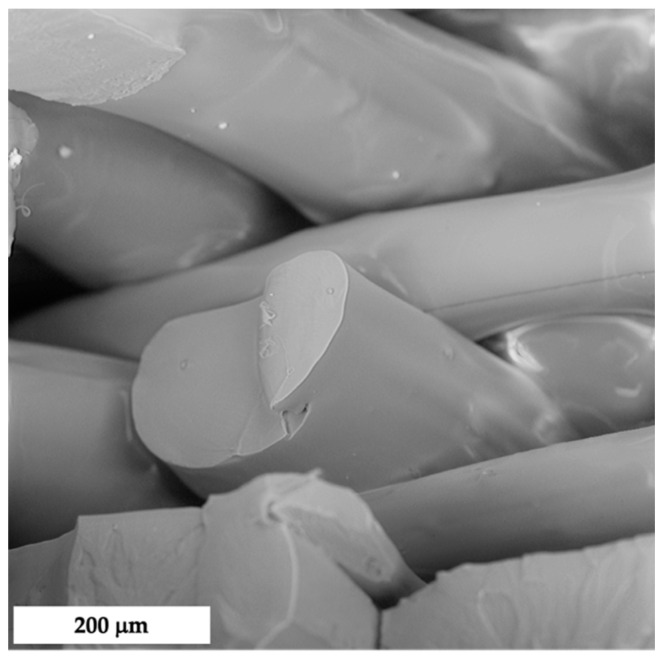
SEM micrograph of fractured cross-section of FDM-PLA.

The morphology of composites prepared by compression-molding is reported in Figure 4, whereas those prepared via FDM are provided in Figure 5. As one can see, the former technique was found to be inadequate to grant satisfactory levels of filler dispersion and adhesion, with the presence of aggregates and voids, due to a lack of mutual affinity. On the contrary, FDM showed suitability to achieve dense structures with enhanced wettability of the fillers, as already observed with another polymeric matrix [6]. In Figure 6, it is possible to notice the peculiar structure of FDM samples induced by the selected raster angle (±45°).

**Figure 7:**
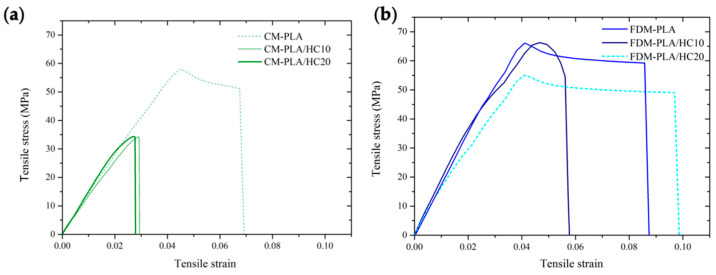
Representative stress–strain curves of CM- (**a**), and FDM-series composites (**b**) during tensile tests.

Representative tensile stress–strain curves of the materials are provided in Figure 7, whereas their salient tensile features are listed in Table 3.

The shape of the tensile curves allows envisaging that pure PLA and FDM-biocomposites (Figure 7b) experience a ductile fracture, owing to the presence of a well-defined necking, otherwise not observed in the case of CM-biocomposites (Figure 7a), which displayed a fragile mode of fracture. Moreover, it can be easily noted that the samples prepared by FDM are much more ductile and resistant than their counterparts prepared by CM. The quantitative analysis of tensile moduli indicated that the choice of processing technique affects the tensile properties of the neat matrix, with CM-PLA being stiffer than FDM-PLA. This aspect could be reasonably explained by considering the eventual differences imparted by the type of printing, either in terms of porosity, surface texture, or degradation.

**Figure 8:**
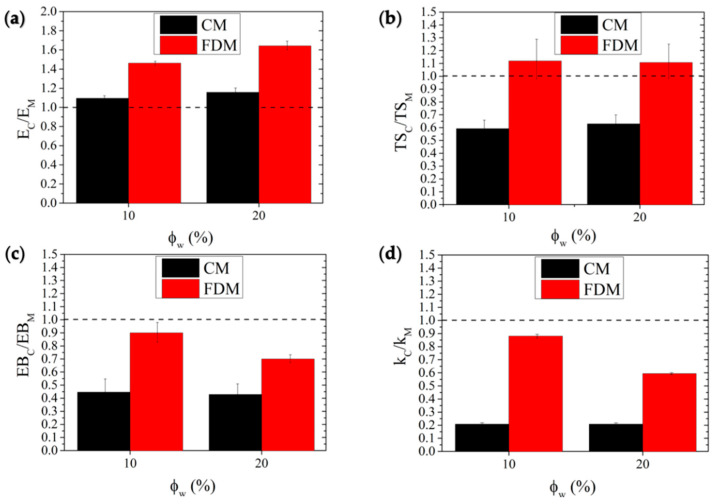
Elastic modulus (**a**), tensile strength (**b**), elongation at break (**c**), and toughness (**d**) of composites normalized with respect to those of matrix. Dashed lines refer to neat matrix value of the respective reduced property.

Furthermore, the effect of filler loading was found to be quite different, depending on the fabrication technique adopted. In fact, although the incorporation of HC imparted a relevant stiffening effect in all the cases, the relative increments achieved were higher in FDM-samples, as visible by comparing the reduced moduli of CM- and FDM-series systems (Figure 8a), i.e., normalized to that of the respective processed matrix. The analysis of breaking properties is reported in Figure 8b,c. With regard to reduced tensile strength (Figure 8b), it can be observed that filler incorporation exerts a fair strengthening effect in FDM-biocomposites (10% more resistant than neat matrix), while being detrimental in CM-biocomposites, which experienced a 35–40% loss in TS with respect to PLA, likely due to the premature failure of specimens. In fact, although all the biocomposites proved less deformable than the corresponding matrices, a more remarkable decay upon filler loading was observed in CM-biocomposites that retained less than half of the matrix EB (Figure 8c). Toughness (Figure 8d) followed the same trend as EB, with all the biocomposites displaying a lower ability to absorb energy and plastically deform without fracturing, when compared to the corresponding matrices. However, even in this case, FDM biocomposites retained 60–90% of the initial toughness of PLA, while CM biocomposites showed toughness values equal to only 20% of that of PLA.

**Figure 9:**
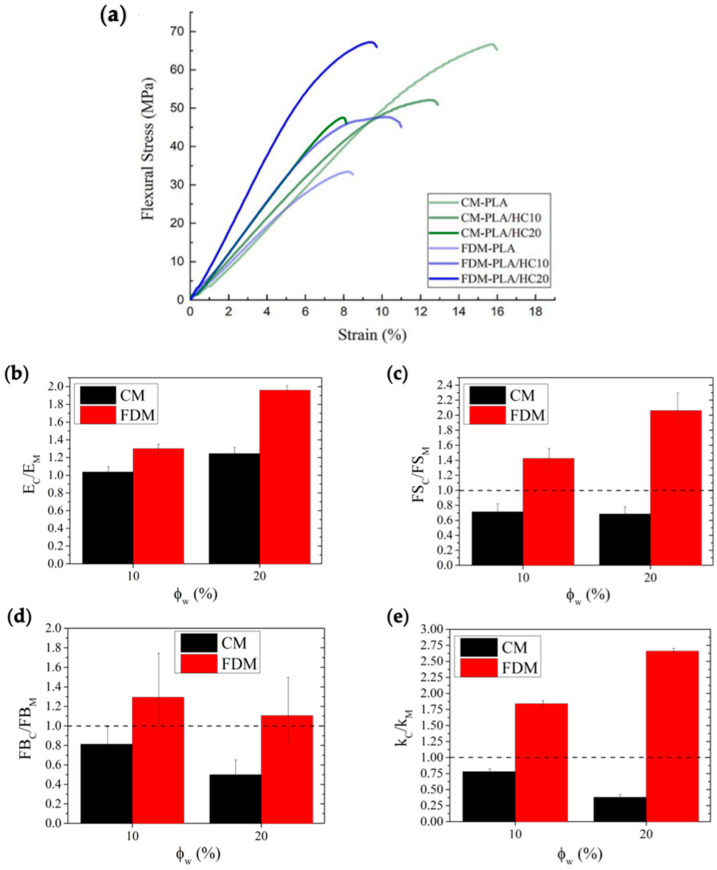
Representative flexural stress–strain curves (**a**), along with behavior of reduced flexural elastic modulus (**b**), strength (**c**), deformability (**d**), and toughness (**e**), as a function of filler content and processing technique. Dashed lines refer to neat matrix value of the respective reduced property.

Figure 9 reports the representative stress-strain curves of the materials subjected to flexural testing (Figure 9a), along with the behavior of reduced flexural properties, namely elastic modulus (Figure 9b), flexural strength (Figure 9c), deformability (Figure 9d), and toughness (Figure 9e), as a function of filler content and processing technique, whereas salient data are listed in Table 4. Even in this type of solicitation, the behavior of neat polymer was greatly affected by the processing technique, although an opposite trend was observed with respect to tensile testing. In fact, in this case, CM-PLA displayed breaking properties much higher than those of FDM-PLA, while the values of elastic modulus were substantially similar. However, the effect of filler incorporation showed remarkable differences, depending on the different printing modes. FDM-composites displayed a monotonic increase of both flexural stiffness and strength upon filler content and proved to be slightly more deformable than pure matrix, while CM-series composites displayed typical embrittlement upon filler addition, with monotonic decay of both flexural strength and deformation. As a consequence, the toughness (Figure 9e) of FDM-biocomposites increases linearly upon filler content (up to +166% relative increase), whereas that of CM-biocomposites was found to decrease monotonically with the filler content, up to halving at 20% loading.

**Figure 10:**
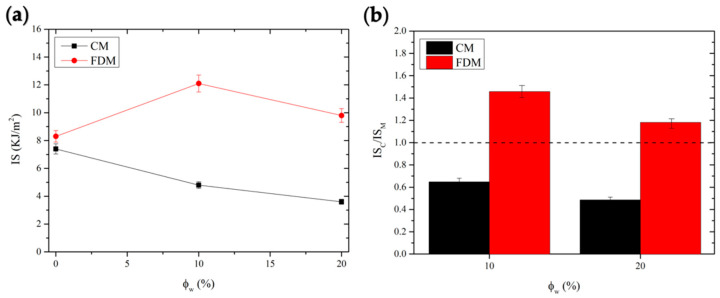
Plots of impact strength (**a**) and reduced impact strength (i.e., normalized to those of each matrix), as a function of filler content and processing technique (**b**). Dashed line refers to neat matrix value of the reduced property.

These outcomes were confirmed in impact properties, measured by Charpy tests, provided in Figure 10. Even in this case, an opposite behavior as a function of filler content was found, depending on the technique used: CM-samples showed a monotonic decrease in impact strength, whereas FDM-composites displayed values higher than those of the pure matrix, with a maximum observed at 10% HC.

Taken together, these outcomes demonstrate that the PLA/HC system is particularly suitable for 3D printing, and leads to materials with a mechanical performance higher than those achieved by compression-molding. Among the biocomposites, FDM-PLA/HC10 gave the overall best performance, while FDM-PLA/HC20 represents the best compromise between mechanical properties and sustainability, given the fact that 20% of PLA can be replaced with inexpensive and renewable biomass fillers, without altering the processing protocol usually adopted for neat polymer.

## 4. Conclusions

Natural fillers achieved using *Hedysarum coronarium* waste were assessed as reinforcing agents for polylactic acid-based biocomposites, prepared by twin-screw extrusion and printed by either FDM or compression-molding. The materials were fully characterized from a morphological and mechanical point of view. The results indicate the excellent 3D printability of such biocomposites, which showed uniform dispersion of the filler throughout the matrix and a strong interfacial adhesion, and which led to remarkable improvements of all the mechanical properties, when compared to either neat matrix or their compression-molded counterparts. In detail, the most remarkable enhancement was found in the flexural properties, where a doubling of modulus and strength was recorded, followed by tensile resistance and stiffness (up to +60%) and impact strength (+50%), whereas a moderate loss in tensile deformability was observed, especially at the highest loading. Moreover, these formulations, relying on an inexpensive and inedible biomass waste, which can be processed in the same way as neat polymer and in the absence of particular purification/chemically treatment steps, represent an outstanding sustainability, from both an economic and ecological point of view. The encouraging results obtained in this study demonstrate the great potential of such biocomposites in many application fields, including panels for the automotive industry and furniture.

## Figures and Tables

**Table 1:** FDM process parameters.

FDM Operating Parameter	Value
Nozzle temperature	230 °C
Bed temperature	60 °C
Infill rate	100%
Infill pattern	Rectilinear
Raster angle	±45°
Layer thickness of tensile samples	0.1 mm
Layer thickness of flexural/impact samples	0.2 mm
Extrusion width	0.4 mm
Printing speed	20 mm/s
Perimeter shells	1
Sample Orientation	flat

**Table 2:** Formulation of investigated samples.

Sample Code Name	PLA Content (wt%)	HC Content (wt%)	HC Mesh Size (μm)	Production Technique
CM-PLA	100	0	-	CM
CM-PLA/HC10	90	10	<150	CM
CM-PLA/HC20	80	20	<150	CM
FDM-PLA	100	0	-	FDM
FDM-PLA/HC10	90	10	<150	FDM
FDM-PLA/HC20	80	20	<150	FDM

**Table 3:** Main tensile properties of the samples investigated.

Sample	E (MPa)	TS (MPa)	EB (%)	*k* (MJ/m^3^)
CM-PLA	1580 ± 19	54 ± 2	5.6 ± 0.4	2.64 ± 0.01
CM-PLA/HC10	1730 ± 15	32 ± 3	2.5 ± 0.4	0.55 ± 0.01
CM-PLA/HC20	1830 ± 22	34 ± 2	2.4 ± 0.2	0.55 ± 0.01
FDM-PLA	1260 ± 13	56 ± 4	10 ± 0.3	4.09 ± 0.02
FDM-PLA/HC10	1843 ± 20	63 ± 4	9 ± 0.5	3.60 ± 0.02
FDM-PLA/HC20	2070 ± 31	62 ± 3	7 ± 0.1	2.43 ± 0.01

**Table 4:** Main flexural properties of the samples investigated.

Sample	E (MPa)	FS (MPa)	FB (%)	*k* (MJ/m^3^)
CM-PLA	530 ± 10	70 ± 3	16 ± 2.0	5.12 ± 0.04
CM-PLA/HC10	550 ± 10	50 ± 5	13 ± 1.1	3.99 ± 0.04
CM-PLA/HC20	660 ± 25	48 ± 3	8 ± 1.3	1.93 ± 0.02
FDM-PLA	500 ± 10	33 ± 2	8.5 ± 1.2	1.44 ± 0.01
FDM-PLA/HC10	650 ± 10	47 ± 1	11 ± 1.6	2.65 ± 0.01
FDM-PLA/HC20	980 ± 20	68 ± 3	9.4 ± 1.5	3.83 ± 0.02

## Data Availability

The data presented in this study are available on request from the corresponding authors.
